# Electrical conductivity and dielectric behaviour of nanocrystalline La_0.6_Gd_0.1_Sr_0.3_Mn_0.75_Si_0.25_O_3_

**DOI:** 10.1039/c8ra00037a

**Published:** 2018-03-01

**Authors:** Ah Dhahri, E. Dhahri, E. K. Hlil

**Affiliations:** Faculté des Sciences, Monastir, Université de Monastir Avenue de l'environnement 5019 Monastir Tunisia; Laboratoire de Physique Appliquée, Faculté des Sciences de Sfax, Université de Sfax 3000, BP 1171 Tunisia Dhahri.ahmad@gmail.com +216 20 20 45 55; Institut Néel, CNRS et Université Joseph Fourier B.P. 166 38042 Grenoble France

## Abstract

An La_0.6_Gd_0.1_Sr_0.3_Mn_0.75_Si_0.25_O_3_ ceramic was prepared *via* a solution-based chemical technique. X-ray diffraction study confirms the formation of the compound in the orthorhombic structure with the *Pnma* group space. Dielectric properties have been investigated in the temperature range of 85–290 K with the frequency range 40 Hz to 2 MHz. The conductivity spectra have been investigated by the Jonscher universal power law: *σ*(*ω*)  =  *σ*_dc_  +  *Aω*^*n*^, where *ω* is the frequency of the ac field, and *n* is the exponent. The deduced exponent ‘*n*’ values prove that a hopping model is the dominating mechanism in the material. Based on dc-electrical resistivity study, the conduction process is found to be dominated by a thermally activated small polaron hopping (SPH) mechanism. Complex impedance analysis (CIA) indicates the presence of a relaxation phenomenon and allows us to modelize the sample in terms of an electrical equivalent circuit. Moreover, the impedance study confirms the contribution of grain boundaries to the electrical properties.

## Abstract 2

A complex impedance spectrum for La_0.6_Gd_0.1_Sr_0.3_Mn_0.75_Si_0.25_O_3_ sample at different temperatures with electrical equivalent circuit.

## Graphical abstract

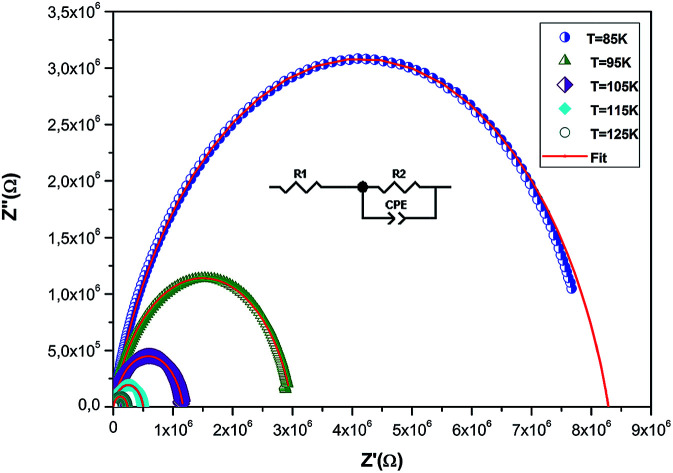

## 1. Introduction

The exploration of properties of inorganic materials has been a long-standing goal in the development of functional materials. There exists a close relationship among structure, morphology and physical properties. Extensive studies have been devoted to understanding the formation mechanisms from both theoretical and experimental point of views to synthesize better functional materials. Perovskite manganites have been the subject of intense research efforts; they exhibit a variety of electronic transport, magnetic and magnetocaloric properties. These properties open up a large field of applications^1,2^. In recent years, several studies have been reported for the dielectric properties of these materials such as of Li_3_(Mg_0.95_A_0.05_)_2_NbO_6_,^3^ CaCu_3_Ti_4_O_12_,^4^ Li_2_Mg_4_TiO_7_ (ref. 5) and Sr_0.5_Ca_0.5_TiO_3_:*x*Pr^3+^ (ref. 6) ceramics. Perovskite manganite materials are present in several technological applications. In the last few years, various studies^7,8^ have suggested that doping at the Mn site influences the polaronic transport. The effect of substituting Mn by Ga, Ru, Fe, Co, Cr and Ti is well studied.^9–14^ The physical properties are successfully explained by a double-exchange (DE) mechanism based on a strong exchange interaction between Mn^4+^ and Mn^3+^ ions through intervening filled oxygen 2p states. It is believed that the interaction between the pairs of Mn^4+^ and Mn^3+^ ions is responsible for the electrical properties in these manganese oxides.^15^

Recently, direct current (dc) and alternating current (ac) transport processes have been investigated in low-hole-doped Ln-based manganite-type perovskites such as La_1−*x*_Ca_*x*_MnO_3_,^16^ La_1−*x*_Sr_*x*_MnO_3_,^17^ La_1−*x*_A_*x*_Mn_1−*y*_Fe_*y*_O_3_,^18^ La_0.7_Sr_0.25_Na_0.05_Mn_0.9_Ti_0.1_O_3_,^19^ La_0.5_Ca_0.5−*x*_Ag_*x*_MnO_3_,^20^ Pr_0.67_A_0.33_MnO_3_ (ref. 21) and RMnO_3_ (R = Eu; Gd; Tb; and Dy),^22^ where manganese is in a mixed valence state (Mn^3+^–Mn^4+^). Pairs of Mn^4+^ and Mn^3+^ can be controlled by changing the doping level or oxygen stoichiometry. Therefore, it seems plausible that the doping element and its content will be important for the electrical properties in these materials. To optimize the properties of this material, a systematic study of the electrical properties should be conducted over a wide temperature range and with different doping levels. Therefore, several investigations have been carried out to understand the correlation among the structure, magnetic and electrical properties, and the magnetoresistance of Re_1−*x*_M_*x*_MnO_3_ by doping with elements such as Cr, Ni, V, Ga, Co, Mg and Al at the Mn site.^23–25^ However, doping of silicon at the Mn site in Re_1−*x*_M_*x*_MnO_3_ (where Re = La, Nd, Pr, Sm, Y, … and M = Pb, Ba, Sr, Ca, …) has not been investigated so far. Therefore, to better understand the role of Mn and its local environment in La_0.6_Gd_0.1_Sr_0.3_MnO_3_, we have studied the effects of replacing some of Mn with Si. To decrease the critical temperature *T*_c_ (*T*_c_ = 378 K)^26^ of the parent compound La_0.7_Sr_0.3_MnO_3_, we have substituted lanthanum (La) with 10% gadolinium (Gd). The choice of the ion Si^4+^ is based on the fact that the ionic radius (0.4 Å) is smaller than that of Mn^4+^ (0.53 Å). In view of this information, the authors have taken up the present research with an objective to study the effect of silicate at the Mn site on the transport and dielectric properties of La_0.6_Gd_0.1_Sr_0.3_Mn_0.75_Si_0.25_O_3_. In the present study, we investigate the dielectric and electrical properties of La_0.6_Gd_0.1_Sr_0.3_Mn_0.75_Si_0.25_O_3_ at different temperatures (77 K to 320 K) and frequencies (40 Hz to 10 MHz). Impedance spectroscopy is successfully employed to obtain clear information about the transport and dielectric properties of this material. Such technique can resolve the conduction components from polycrystalline electrical ceramics, particularly in differentiating the transport characteristics in grains and grain boundaries.

## 2. Experimental

A solution based sol–gel method was used to prepare the La_0.6_^3+^Gd_0.1_^3+^Sr_0.3_^2+^(Mn_0.7_^3+^ Mn_0.05_^4+^)Si_0.25_^4+^O_3_^2−^ fine powder. As starting materials, stoichiometric amounts of La(NO_3_)_3_·6H_2_O (2.5981 g, 6 mmol); Sr(NO_3_)_2_·6H_2_O (0.9592 g, 3 mmol); Mn(NO_3_)_2_·4H_2_O (1.8826 g, 7.5 mmol); Gd(OOCCH_3_)_3_·*x*H_2_O (0.3344 g, 1 mmol); and SiCl_4_ (0.4247 g, 2.5 mmol) were used owing to their high solubility in water. These precursors were weighed in the desired proportions and dissolved with small amounts of deionized water. Citric acid was used as a polymerization/complexation agent, forming a stable solution. Metallic salt solution (100 mL) was added to 300 mL of a solution containing a mixture of citric acid (60 g) and ethylene glycol (13 mL), which dispersed the cations homogeneously forming a cation–polymer network. This stable solution was then heated on a thermal plate under constant stirring where polymerization occurred in the liquid solution and led to a homogeneous sol. This solution was then heated at a temperature of ∼100–140 °C till a dry thick brown-coloured sol was formed. The resin was further decomposed in an oven at a temperature of ∼300 °C in air for 3 h to get a fairly porous polymeric precursor in the form of a black resin-like material. After milling in an agate mortar, the precursor was calcined at 600 °C for 7 h to give a fine powder. Then, the obtained powder was pressed into circular pellets under 4 t cm^−2^ (to about 2 mm thickness) and finally sintered in air at 800 °C for 10 h. The sample was proved to be single phase by powder X-ray diffraction experiments performed at room temperature using a Siemens D-5000 with a graphite monochromatized CuKα radiation (*λ* = 1.54056 Å). The data collection was performed by step-scan modes in a 2*θ* range between 20° and 90° with a step-size of 0.0167° and a count time of 18 s per step. This system was able to detect up to a minimum of 3% of impurities according to our measurements. The structural parameters were obtained by fitting the experimental data from XRD using the Rietveld structural refinement program FULLPROF soft-ware (Version 1.9c-May 2001-LLB-JRC).^27^ The compound was pressed into polished pellets with a diameter of 10 mm and a thickness of about 1 mm and then sintered. For electrical measurements, two indium plots separated by a distance of 5 mm were deposited on the pellets to ensure ohmic contact. The other side was bound to the cold finger of liquid nitrogen cooled cryostat to vary the sample temperature between 77 and 320 K. The two indium pads were connected to the electrodes of an Agilent 4294A impedance analyzer to measure the sample conductance. The samples were modeled with a parallel circuit and excited by an alternating signal with an amplitude of 50 mV.

## 3. Results and discussion

### 3.1 Microstructure analysis

**Fig. 1:**
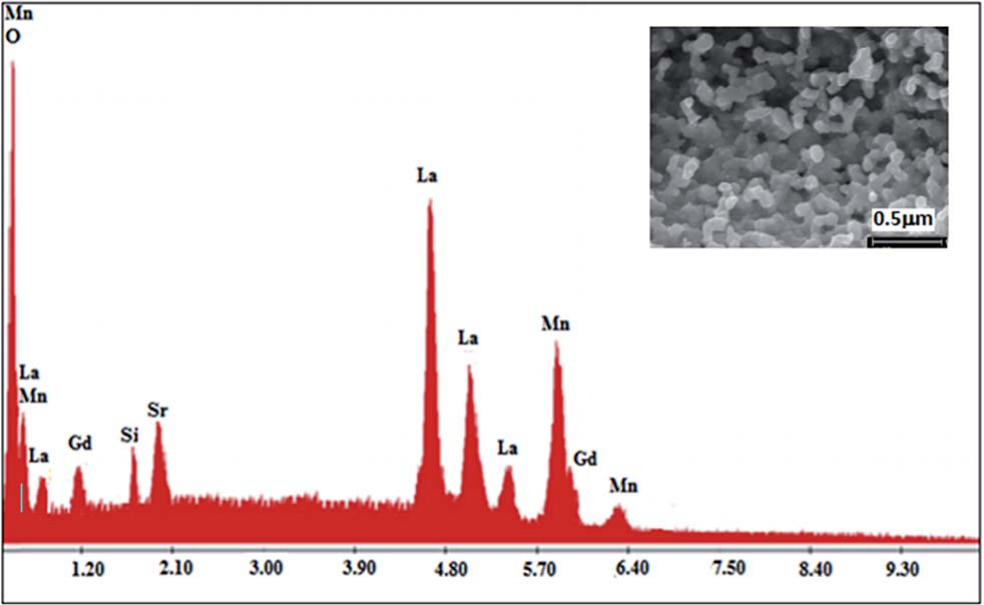
EDX spectra of the La_0.6_Gd_0.1_Sr_0.3_Mn_0.75_Si_0.25_O_3_ compound. The inset shows a typical scanning electron micrography (SEM) image.

To check the existence of all the elements in the La_0.6_Gd_0.1_Sr_0.3_Mn_0.75_Si_0.25_O_3_ (LGSMSiO) compound, energy dispersive X-ray analysis was performed. The EDX spectra shown in Fig. 1 reveals the presence of the elements La, Sr, Gd, Si, and Mn, which confirmed that there was no loss of any integrated elements during the sintering. The EDX analysis showed that the chemical composition of the compound was close to that of the nominal one (La : Gd : Sr : Mn : Si = 0.6 : 0.1 : 0.30 : 0.75 : 0.25). The typical cationic composition for the sample is represented in Table 1. The result was very close to that of the nominal one within experimental uncertainties, and since no secondary phases were seen in the XRD patterns, it was reasonable to assume that silicon (Si) had been substituted for Mn in this sample.

**Table 1:** Results of EDX analysis

Typical La	Cationic Gd	Composition Sr	From Mn	EDX Si	Nominal composition
0.61	0.11	0.31	0.74	0.26	La_0.6_Gd_0.1_Sr_0.3_Mn_0.75_Si_0.25_O_3_

The SEM image of the fractured surface of the Si-doped sample (inset of Fig. 1) reveals the presence of a large distribution of grains that connect with each other. The average grain size in the sample is estimated to be about ∼120–126 nm. The presence of the microstructural characteristics can be related to the matter transport mechanism between the grains during the sintering process.

**Fig. 2:**
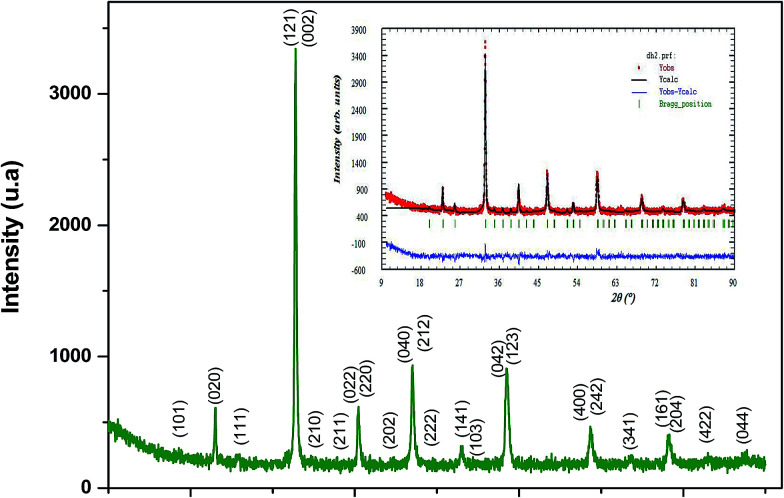
X-ray diffraction patterns of the La_0.6_Gd_0.1_Sr_0.3_Mn_0.75_Si_0.25_O_3_ compound at room temperature. All the peaks of the manganite phase are indexed in the orthorhombic *Pnma* symmetry. The inset shows observed (symbol) and calculated (solid line) XRD patterns obtained by the Rietveld analysis for the manganite. Their difference (enlarged scale) is represented at the bottom of the figure (solid line). The vertical ticks show the positions of the calculated Bragg reflections for the orthorhombic phase (*Pnma* space group).

The average crystallite size values have been estimated from the full width at half maxima of the X-ray diffraction peaks. The effects of synthesis and instrumental and processing conditions have been taken into consideration while making the calculation of crystallite sizes. The broadening of the Bragg reflections due to micro strains is considered to have angular dependence and is given by: *β*_strain_ = 4*ε* tan *θ*, where *β*_strain_ is the peak shift due to the strain, 
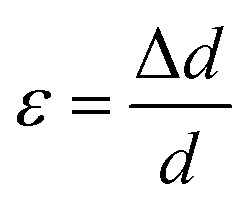
 is a coefficient related to the strain and *θ* is the Bragg angle. The micro strains include the effects of structural defects such as dislocations, stacking faults, twin boundaries, and inter growths. The dependence of the size effect is given by the Scherer formula: 
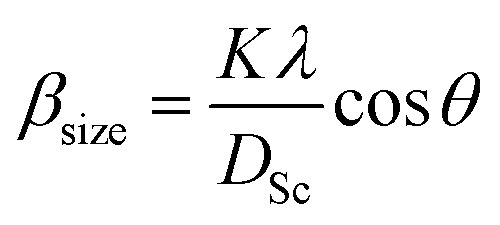
, where *K* is the grain shape factor (for a spherical grain *K* = 0.89), *λ* is the wavelength of the X-rays used (*λ* = 1.5406 Å), *D*_Sc_ is the crystal thickness and *θ*_max_ is the corresponding incident angle. The value of (*D*_Sc_) is 30 nm. In the present investigation, only the prominent peaks have been considered. The instrument broadening effect has been eliminated by subtracting the full width at half maxima (*β*_0_) values from the *β* size at respective Bragg peaks of a standard Si sample. Finally, the complete expression for the full width at half maximum (FWHM) of the X-ray diffraction peaks is given by: 
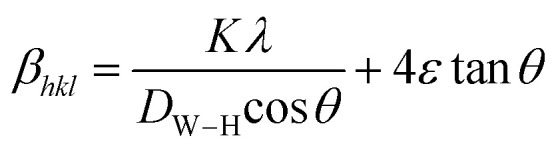
. A plot between *β*_*hkl*_ cos *θ* and sin *θ* gives a straight line, and from the value of the intercept on the *y*-axis, the average crystal size is calculated and is found to be 45 nm. The crystallite size, calculated in the present system using the Williamson–Hall technique, is larger as compared to the crystallite size from the Debye–Scherer method (*D*_Sc_) because the broadening effect due to the strain (*ε* = 0.14%) is completely excluded in the Debye–Scherer technique. Obviously, the particle sizes observed by SEM are several times larger than those calculated by XRD, which indicates that each particle observed by SEM consists of several crystallized grains.^28^Fig. 2 shows the X-ray diffraction patterns of LGSMSiO at room temperature. The sample is single phase without detectable secondary phases within the sensitivity limits of the experiment (a few percent). The structure refinement is performed in the orthorhombic setting of the *Pnma* (*Z* = 4) space group (no. 62) (inset of Fig. 2) in which the (La/Gd/Sr) atoms are at 4*c* (*x*, 0.25, *z*) position, (Mn/Si) atoms are at 4*b* (0.5,0,0) position, O(1) is at 4*c* (*x*,0.25, *z*) position and O(2) is at 8*d* (*x*, *y*, *z*) position. Table 2 summarizes the relevant structural parameters obtained by the Rietveld analysis of the powder XRD pattern. This table also reports the residuals for the weighted pattern *R*_wp_, the pattern *R*_p_, the structure factor *R*_F_ and the goodness of fit *χ*^2^. The tolerance factor, which is the geometric measure of size mismatch of the perovskite: 
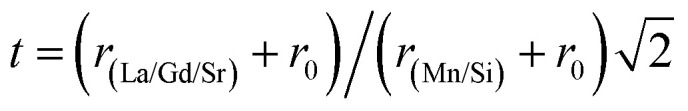
, is equal to 0.95 and is in the stable range for the perovskite structure 0.75 < *t* < 1.02.^29^

**Table 2:** Crystallographic data from the Rietveld refinement of X-ray diffraction data for the La_0.6_Gd_0.1_Sr_0.3_Mn_0.75_Si_0.25_O_3_ sample^a^

LGSMSiO	Space group	*Pnma*
Cell parameters	*a* (Å)	5.4563(2)
	*b* (Å)	7.7241(3)
	*c* (Å)	5.4961(5)
	*V* (Å^3^)	231.63
Atoms	La/Gd/Sr*x*	0.0031(6)
	*z*	0.0046(2)
	*B*_iso_ (Å^2^)	0.32(3)
	Mn/Si *B*_iso_ (Å^2^)	0.26(3)
	(O_1_)*x*	0.4837(2)
	*y*	0.963(6)
	*B*_iso_ (Å^2^)	1.75(2)
	(O_2_)*x*	0.31(1)
	*y*	0.0019(5)
	*z*	0.681(3)
	*B*_iso_ (Å^2^)	1.82(2)
Structural parameters	*d*_Mn–O_1__ (Å)	1.966(4)
	*θ*_Mn–O_1_–Mn_ (°)	160.12(2)
	*d*_Mn–O_2__ (Å)	1.962(1)
	*θ*_Mn–O_2_–Mn_ (°)	166.88(3)
	〈*d*_Mn–O_〉 (Å)	1.964(4)
	〈*θ*_Mn–O–Mn_〉 (Å)	163.50(5)
Density (theo.) (g cm^−3^)		6.35
Density (exp.) (g cm^−3^)		6.223
Compactness: *C*		0.98
Agreement factors	*R*_p_ (%)	3.55
	*R*_wp_ (%)	2.12
	*R*_F_ (%)	2.75
	*χ* ^2^	1.65

a Density (exp.): 
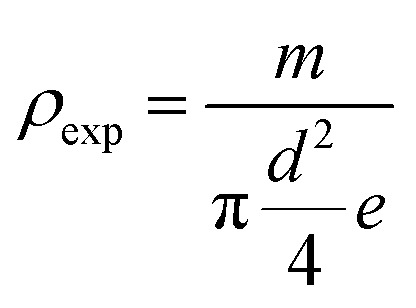
. Density(theor.).

The percentage of orthorhombic deformation, *D*%, can be obtained using the formula:^30^
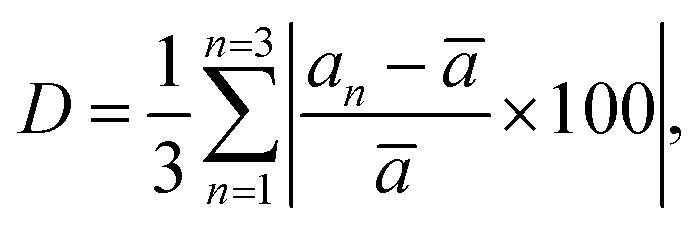
where *a*_1_ = *a*, *a*_2_ = *b*,
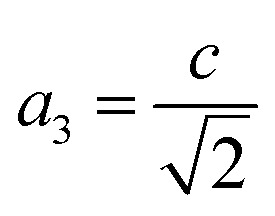
, 
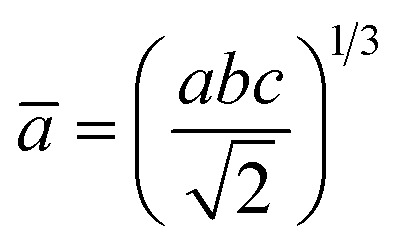
 and *a*, *b*, *c* are the lattice parameters. The *D*% value for the sample estimated by using this equation is found to be 4%.

The experimental density is determined from the weight and geometrical dimensions of the cylindrical pellets and then compared to the theoretical density. The compactness is thus calculated as the ratio. Table 2 shows the values of *ρ*_exp_, *ρ*_theor_ and *C*.

### 3.2 Electrical conductivity analysis

Electrical conductivity in the materials is a thermally activated process that takes place due to the ordered motion of weakly bound charged particles under the influence of an electric field. It is one of the significant properties of the materials to be characterized and depends on the nature of charge carriers that dominate the conduction process, such as electron/holes or cations/anions and their response as a function of temperature and frequency. For the LGSMSiO materials, the electrical conductivity is mainly due to the hopping of electrons between the ions of the same element that are present in more than one valence state (Mn^3+^ → Mn^4+^).^31^ The charges can migrate under the influence of the applied field and contribute to the electrical response of the system. The frequency dependence of the conductivity generally obeys Jonscher's power law:^32^

1 *σ*(*ω*)  =  *σ*_dc_  +  *Aω*^*n*^

where *σ*(*ω*) is the total conductivity, *σ*_dc_ is the direct current conductivity of the sample, *Aω*^*n*^ is the pure dispersive component of AC conductivity having a characteristic of power law in terms of angular frequency *ω* and exponent *n* (0 ≤ *n* ≤ 1) that represents the degree of interaction between mobile ions and the lattices around them, and *A* is a constant which determines the strength of polarizability.

**Fig. 3:**
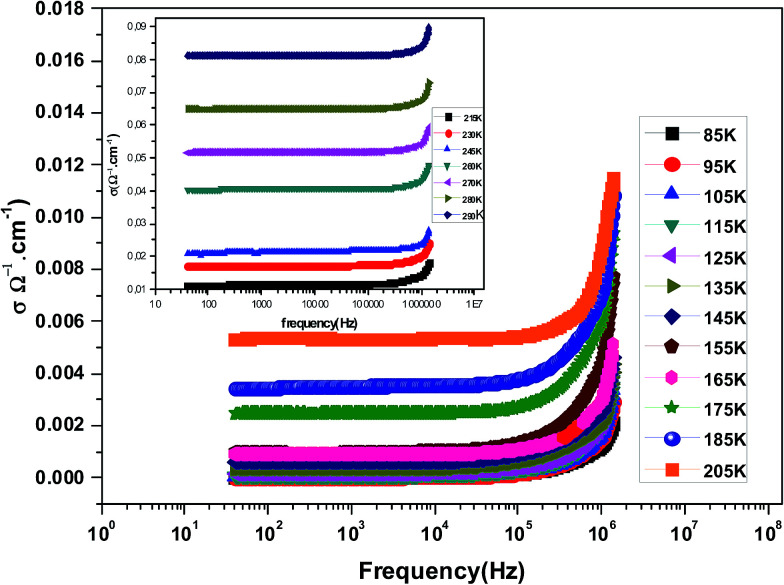
The variation at room temperature of the ac conductivity (*σ*) *versus* frequency for La_0.6_Gd_0.1_Sr_0.3_Mn_0.75_Si_0.25_O_3_.

**Fig. 4:**
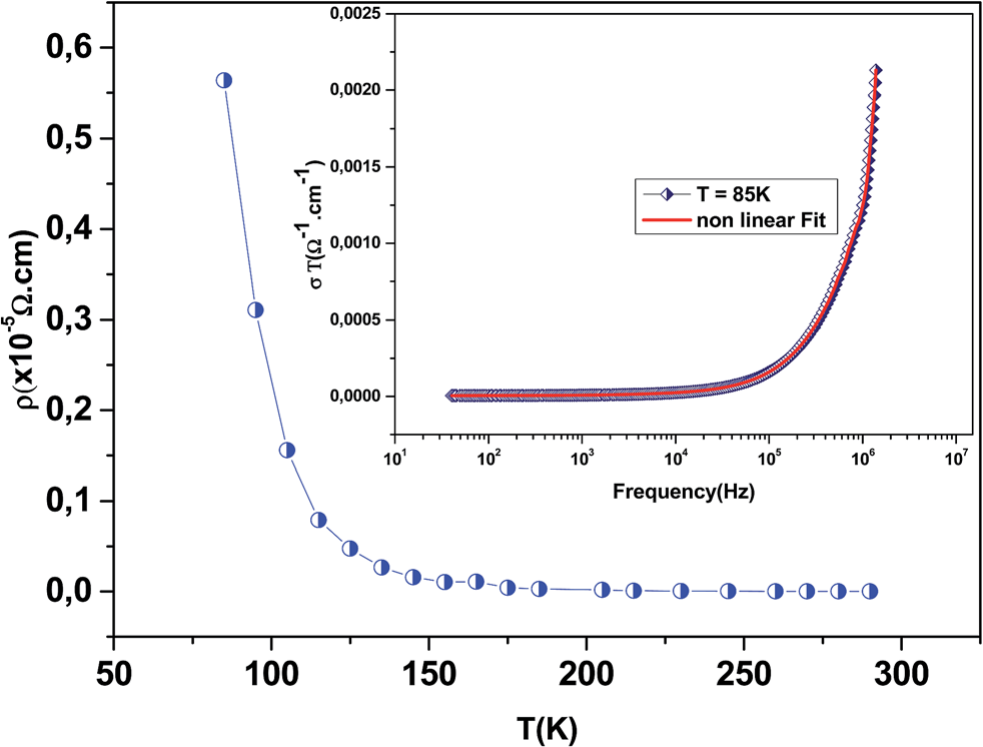
The variation of the electrical resistivity (*ρ*) *versus* temperature deduced from the dc conductivity *σ*(0). The inset shows the non-linear fitting (red solid line) of conductivity (*σ*_T_) obeying the Jonscher power law for La_0.6_Gd_0.1_Sr_0.3_Mn_0.75_Si_0.25_O_3_ at *T* = 85 K.

**Fig. 5:**
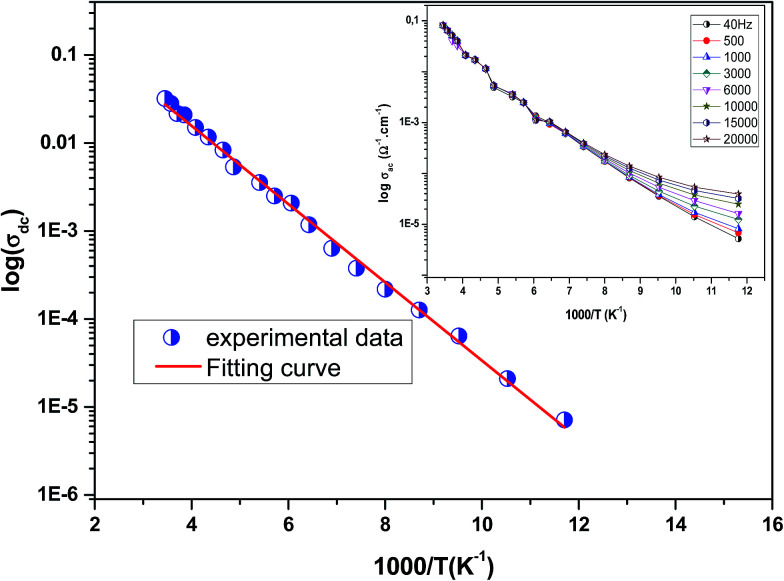
Bulk conductivity for the La_0.6_Gd_0.1_Sr_0.3_Mn_0.75_Si_0.25_O_3_ sample as a function of temperature. Inset shows the variation of *σ*_ac_ conductivity with temperature at different frequencies of the La_0.6_Gd_0.1_Sr_0.3_Mn_0.75_Si_0.25_O_3_ compound.

Fig. 3 shows the variation of AC conductivity as a function of frequency at different temperatures of the La_0.6_Gd_0.1_Sr_0.3_Mn_0.75_Si_0.25_O_3_ sample. It is characterized by the following points: (i) a plateau region at low frequencies corresponds to *σ*_dc_. In this frequency region, the conductivity, *σ*_dc_, increases with the increasing temperature. Such behavior indicates that the electrical conductivity in the material is a thermally activated process. (ii) At high frequency, the conductivity is governed by *Aω*^*n*^, where *n* is a constant and *ω* is the angular frequency. According to Jonscher, the origin of the frequency dependence of conductivity can be due to the relaxation phenomena of the ionic atmosphere arising from the mobile charge carriers.^33^ The experimental conductivity spectra of the sample are fitted using eqn (1). The fitting results are summarized in Table 3. From this table, we can conclude that the exponent *n* increases with the increasing temperature. This change of *n* with temperature corresponds to a thermally activated process. The temperature dependence of *n* gives information to specify the suitable mechanism involved for the AC conductivity. In most cases, the frequency exponent, *n*, is found to be between 0.6 and 1 for ionic conducting compounds.^34^ In our study, *n* is lower than 1 for temperatures lower than *T* = 205 K. This can be attributed to the hopping conduction of mobile charge carriers over the barrier between two sites, which is similar to that observed in amorphous semiconductors and glasses.^35^ However, for temperatures above 205 K, *n* is larger than one. This may be attributed to the motion of mobile charge carriers from site to site with quantum mechanical tunneling between asymmetric double-well potentials as is proposed by K. S. Gilroy *et al.*^36^ The inset of Fig. 4 represents an illustration of this fitting for the temperature *T* = 85 K. It can be seen that the fit (red solid line) matches well with the experimental values. Fig. 4 shows the plots of the electrical resistivity (*ρ*) *versus* temperature deduced from the dc conductivity (*σ*_dc_) using the following relation: 
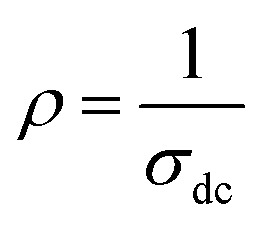
. This curve indicates that this compound exhibits semiconductor behavior across all the studied temperature ranges. Fig. 5 shows the variation of dc conductivity (*σ*_dc_) *versus* 10^3^/*T* used for the calculation of activation energy, and the plot clearly obeys the Arrhenius relation *σ*_dc_  =  *σ*_0_ exp(−*E*_g_/*k*_B_*T*), where *σ*_0_ is the pre-exponential factor corresponding to 1/*T* = 0, *k*_B_ is the Boltzmann constant (=8.617 × 10^−5^ eV K^−1^), *E*_g_ is the conduction activation energy and *T* is the absolute temperature. For the sample, the activation energy (*E*_g_) calculated from the slope of the graph is ∼(0.0383 ± 0.006) eV at 85–290 K.

**Table 3:** The best fitting parameters obtained from experimental data of the conductivity (*σ*) as a function of frequency using the Jonscher's power law

Temperature (K)	*σ*_dc_ (×10^−5^ Ω^−1^ cm^−1^)	*A* (×10^−9^)	*n*	*R* ^2^ (%)
85	1.774	1.099	0.8856	99.99
95	3.218	7.907	0.8932	99.98
105	6.415	6.499	0.9165	99.99
115	12.670	3.785	0.9264	99.99
125	21.015	2.885	0.9322	99.99
135	37.727	5.751	0.9453	99.97
145	63.580	4.939	0.9561	99.99
155	96.416	1.328	0.9643	99.98
165	97.557	1.861	0.9723	99.99
175	250	3.584	0.9801	99.99
185	354	2.641	0.9857	99.99
205	535	4.361	0.9984	99.98
215	1145	7.914	1.0165	99.99
230	1723	2.021	1.0328	99.97
245	2140	7.812	1.0443	99.98
260	4054	9.342	1.0730	99.96
270	5173	5.898	1.1122	99.97
280	6486	3.425	1.1323	99.99
290	8112	2.547	1.2321	99.98

Inset of Fig. 5 shows the variation of *σ*_ac_  with temperature at different frequencies. It was observed that *σ*_ac_  increased with the increase in temperature, and it obeyed the thermally activated relation: *σ*_ac_  =  *σ*_0_ exp(−*E*_g_/*k*_B_*T*), where *σ*_0_ is the pre-exponential factor, *k*_B_ is the Boltzmann constant, *E*_g_ is the activation energy and *T* is the absolute temperature.^37^ It was also seen that the slope of the curve decreased with the increase in frequency. This indicated a reduction in *E*_g_ with increase in frequency due to enhanced electronic jumps between localized states.^37^ Furthermore, a merge of all of the curves at high temperatures may be due to intrinsic conductivity of the material at these temperature regions.^38^

### 3.3 Modulus analysis

**Fig. 6:**
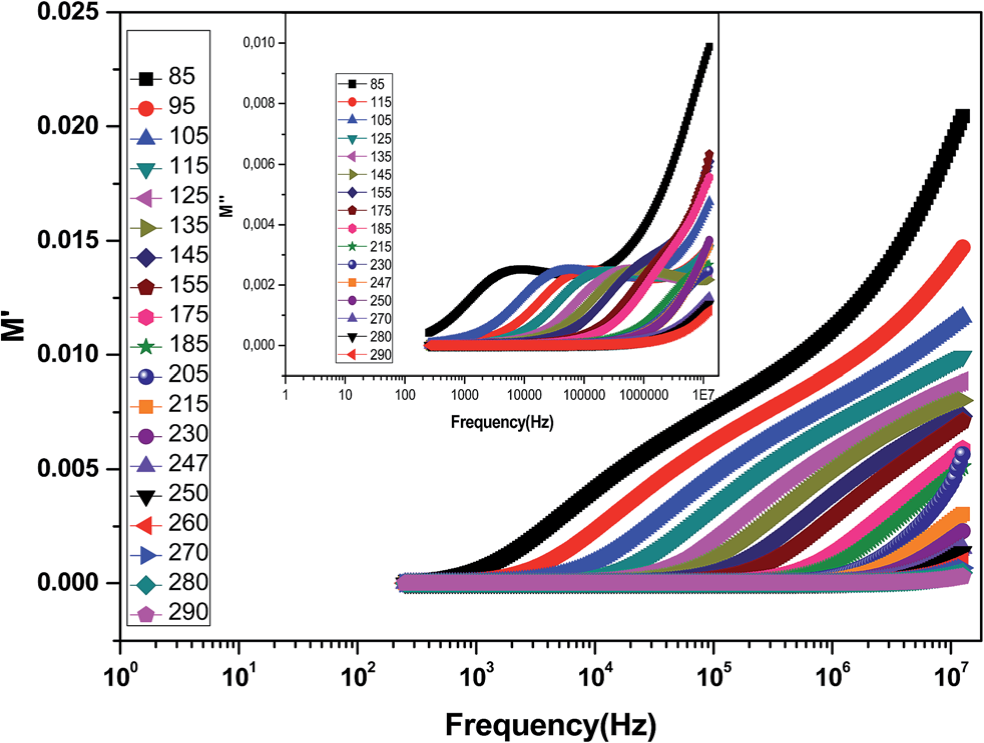
Variation of *M*′ with frequency at different temperatures for the La_0.6_Gd_0.1_Sr_0.3_Mn_0.75_Si_0.25_O_3_ compound. The inset shows the variation of *M*′′ with frequency at different temperatures.

The complex electric modulus formalism can easily distinguish between electrode polarization effects and grain boundary conduction processes. It is also useful in detecting the bulk properties that appear from relaxation time.^39^ The variations of the imaginary part of the electrical modulus ‘*M*’ at different temperatures are shown in the inset of Fig. 6 from which we can note that the positions of the relaxation peaks shift toward higher frequencies as the temperature is increased. The low frequency side of the imaginary part of the modulus determines the range in which the charge carriers are mobile over long distances (the charge carriers suggest the possibility of ion migration *via* hopping from one site to the neighboring site). At a frequency above the peak maximum ‘*M*’ (high frequency), the carriers are spatially confined to potential wells being mobile over short distances and thus can be made to have localized motion within the well. The electric modulus M* represents the real dielectric relaxation process and should be replaced by a frequency dependent electric modulus which can be expressed by the following relation:^40^
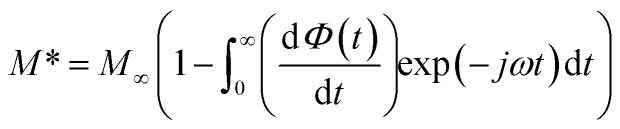
 , where *M*_∞_ = 1/*ε*_∞_ is the asymptotic value of *M*′(*ω*) and *ϕ*(*t*) = exp(−(*t*/*τ*_M_)^*β*^) represents the time evolution of the electric field within the material where *β* (0 < *β* < 1) is the stretched exponent and *τ*_M_ is the conductivity relaxation time.

**Fig. 7:**
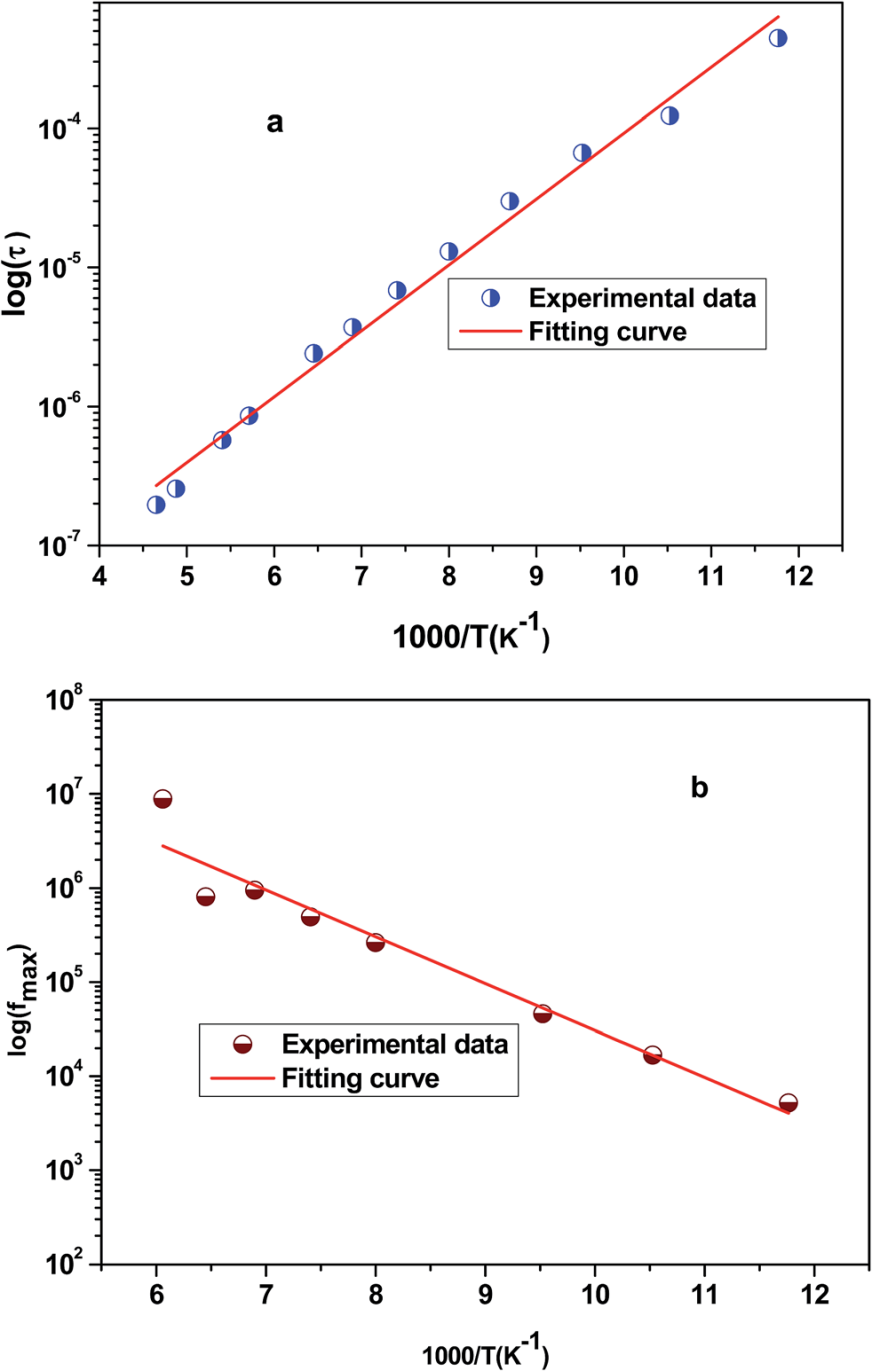
(a) Variation of logarithmic relaxation time log(*τ*) *versus* 10^3^/*T* and (b) plot of log(*f*_max_) *versus* 10^3^/*T*.

Fig. 6 shows the variation of the real part of the electrical modulus ‘*M*’ *versus* frequencies at different temperatures. We can note a very low ‘*M*’ value (close to zero) in the low-frequency region that increases with the increasing frequency, ultimately approaching the *M*_∞_ value. This may be attributed to a conduction phenomenon due to short-range mobility of charge carries. Herein, the relaxation frequency (*f*_max_) corresponding to the ‘*M*’ peak denotes the transition from long-range to short-range motion with the increasing frequency. The temperature dependence of the frequency *f*_max_ in the low-frequency side of the dielectric relaxation can be described by the Arrhenius law. Thus the activation energy (*E*_relax_) is calculated from 
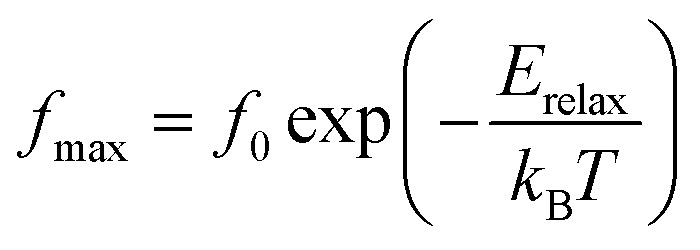
, where *f*_0_ is the pre-exponential term. Fig. 7 presents the variation of the logarithmic relaxation time log(*τ*) *versus* 10^3^/*T* and log(*f*_max_) *versus* 10^3^/*T* in the temperature range of 85–215 K. The main values of the relaxation energy and the relaxation time obtained from the equation *τ*_0_
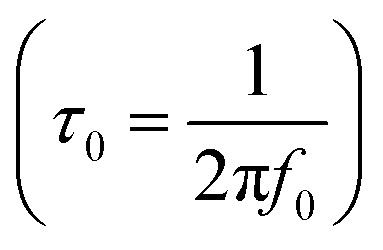
 are 42.92 meV and 1.2 × 10^−9^ s, respectively.

### 3.4 Complex impedance spectroscopy

To understand the effect of substitution of manganese by the non-magnetic Si ion on the electrical properties over a wide range of frequencies and temperatures, impedance spectroscopy was employed. This technique is one of the most useful investigation techniques because the impedance of grains can be separated from the order sources of impedance, namely grain boundaries and electrode effects.^41^ In complex impedance diagrams (Nyquist or Cole–Cole), the imaginary part of the impedance (*Z*′′) was plotted against the real part of impedance (*Z*′). The response of an ideal parallel circuit of resistance and capacitance ‘*C*’ was a semicircle centered on the real axis. *C* was calculated from the frequency of the semicircle maximum, whereas *R* was determined from the diameter of the semicircle. The impedance analysis of a compound is based on an idealized circuit model with discrete electrical components. This analysis is mainly accomplished by fitting the impedance data to an equivalent circuit, which is representative of the material under investigation.

**Fig. 8:**
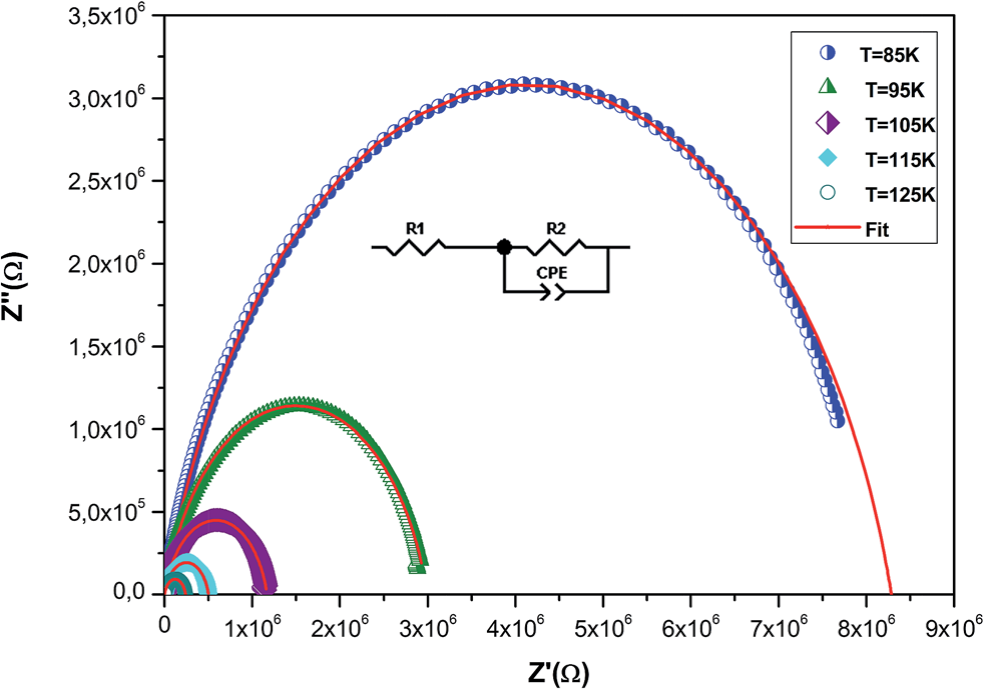
Complex impedance spectrum (Nyquist plot) for the La_0.6_Gd_0.1_Sr_0.3_Mn_0.75_Si_0.25_O_3_ sample at different temperatures with the electrical equivalent circuit (see the inset).

Fig. 8 shows the imaginary part of the impedance (*Z*′′) *versus* the real part (*Z*′) over a wide range of frequencies and at different temperatures. These plots are characterized by the appearance of semicircular arcs whose maxima decease with the increasing temperature. The appearance of a single semicircle at all the temperatures means that the electrical processes obey a single relaxation mechanism.^42^ The diameter of the semicircle decreases with the increasing temperature, demonstrating a pronounced increase in dc conduction. To interpret such a diagram, it is necessary to model the compound.^43,44^ The experimental data are fitted using the Zview software and the best fit (in Fig. 8, it is presented as a red solid line) is obtained when employing an equivalent circuit formed by a resistance *R*_1_ (grain resistance *R*_g_) in series with a parallel combination of a constant phase element impedance (*Z*_CPE_) and resistance *R*_2_ (grain boundary resistance *R*_gb_). The equivalent configuration is of the type (*R*_1_ + (*R*_2_//*Z*_CPE_)) as shown in the inset of Fig. 8. The values of all fitted parameters are tabulated in Table 4. As the grain resistance (*R*_g_) is too weak, the total resistance (*R*_T_), which is the sum of grain and grain boundary resistances, is approximately equal to the grain boundary contribution.^45^

**Table 4:** Electrical parameters of the equivalent circuit deduced from complex impedance spectrum for the La_0.6_Gd_0.1_Sr_0.3_Mn_0.75_Si_0.25_O_3_ compound

*T* (K)	*R*_1_ = *R*_g_ (Ω)	*R*_2_ = *R*_gb_ × 10^5^ (Ω)	CPE (nF)	*α*
85	680.3	8.2857	0.228	0.836
95	658	2.995	0.2347	0.828
105	629	1.1684	0.549	0.825
115	550	0.5005	0.2842	0.821
125	465.3	0.2395	0.3568	0.819
135	461.12	0.1220	0.358	0.819
145	419	0.06760	0.3933	0.817
155	395	0.04220	0.999	0.815
175	384	0.01510	1.285	0.812
185	372	0.0123	1.448	0.806
215	366	0.0106	1.690	0.799
230	357	0.0102	1.978	0.798
245	251	0.0098	2.122	0.789
260	130	0.0085	2.462	0.796
270	92	0.0064	3.025	0.792
280	73	0.0058	3.326	0.798
290	60	0.0049	4.651	0.795

The grain boundary resistance decreases with the increasing temperature, indicating a semi-conducting behavior for the compound (Table 4). This result is in good agreement with that of *ρ*(*T*) curves (Fig. 4). Such behavior has also been reported in other studies.^46^ It is revealed that the effect of grain boundaries helps lower the barrier to movement of the load carriers, resulting in increased electrical transport with an increase in temperature.

**Fig. 9:**
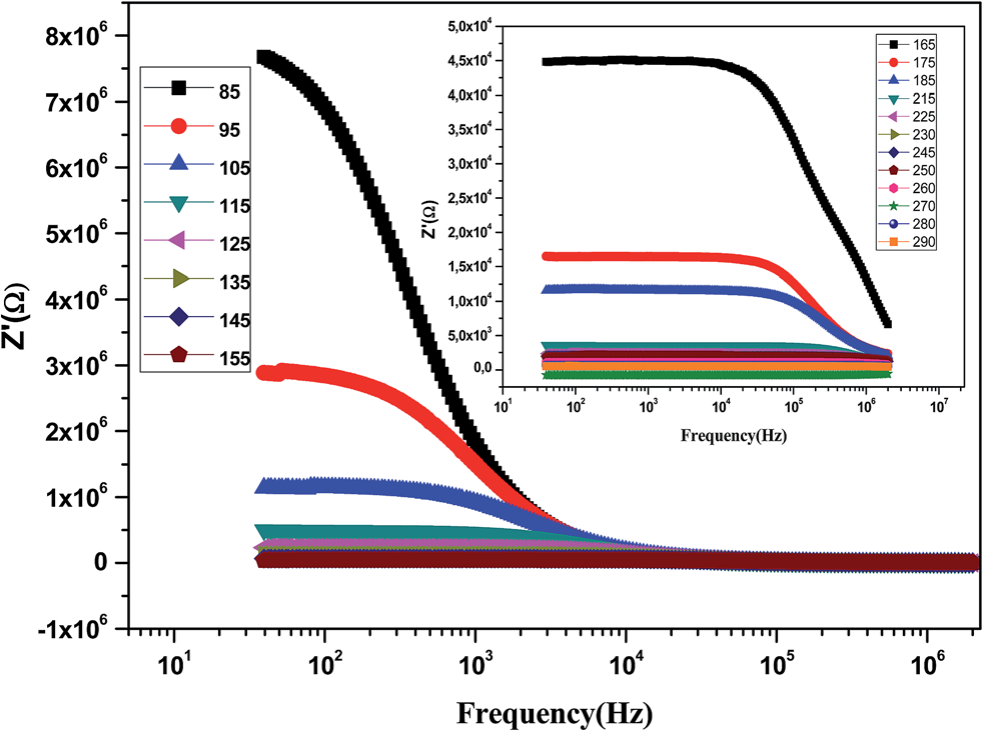
Variation of the real part of the impedance (*Z*′) of the La_0.6_Gd_0.1_Sr_0.3_Mn_0.75_Si_0.25_O_3_ sample as a function of frequency for different temperatures.

Fig. 9 shows the variation of the real part of impedance (*Z*′) with frequency at different temperatures. It is clear from the *Z*′ = *f*(freq) curves that the impedance value is higher at lower temperatures in the low-frequency domain and then, it decreases gradually with the increasing frequency. It is seen that *Z*′ decreases with an increase in frequency, which signifies the enhancement of ac conductivity with the increase in frequency. Moreover, *Z*′ decreases with an increase in temperature. It is observed that *Z*′ values merge at the high frequency side, which is due to a possible release of space charge.^47^

**Fig. 10:**
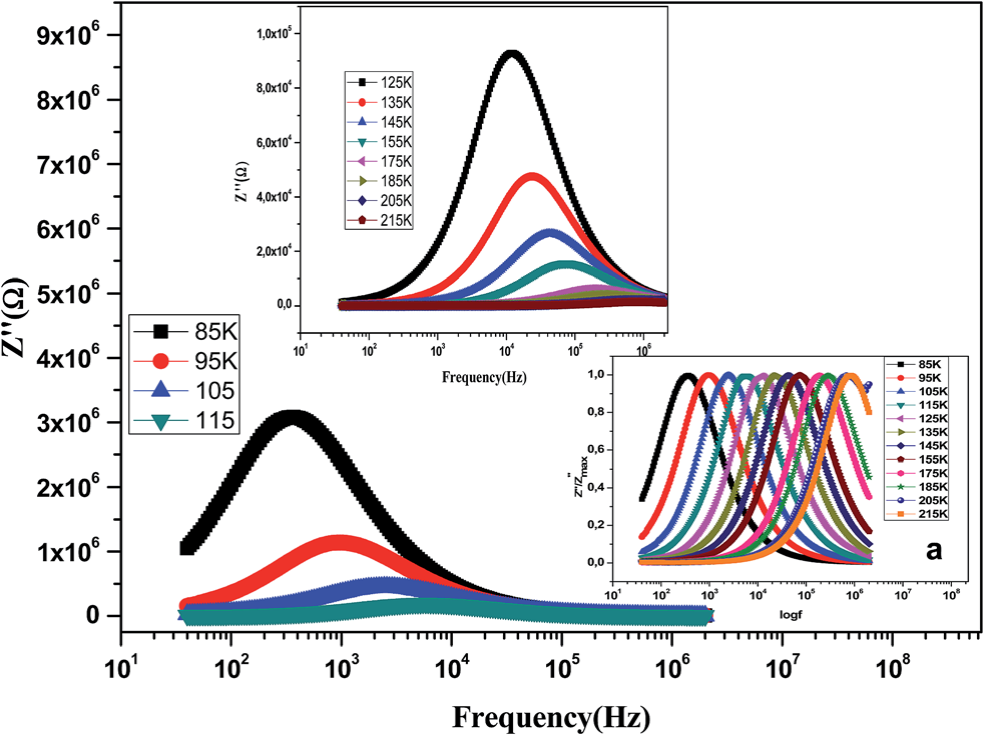
Variation of the imaginary part of the impedance (*Z*′′) of the La_0.6_Gd_0.1_Sr_0.3_Mn_0.75_Si_0.25_O_3_ compound as a function of frequency for different temperatures. Inset: variation of 
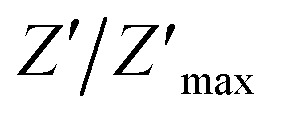
 with log(*f*).

The variation of the imaginary part of impedance (*Z*′′) of the La_0.6_Gd_0.1_Sr_0.3_Mn_0.75_Si_0.25_O_3_ sample as a function of frequency for different temperatures is shown in Fig. 10. It is found that the *Z*′′ value decreases with the increase in both temperature and frequency, and it shifts to higher frequencies as the temperature increases. This behavior describes both the type and the strength of the thermal relaxation processes in the material. This process is probably due to the presence of electrons and/or immobile species at lower temperatures and defects and vacancies at higher temperatures.^47^ The inset in Fig. 10a shows the normalized imaginary parts of the impedance 
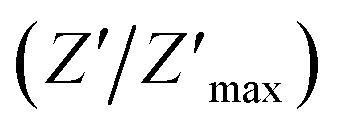
 as a function of frequency at the selected temperatures. The peaks are observed with a slight symmetric broadening at each temperature, especially at higher temperature. The asymmetric broadening of the peaks suggests the presence of electrical processes in the material with a spread of relaxation times.^48^

**Fig. 11:**
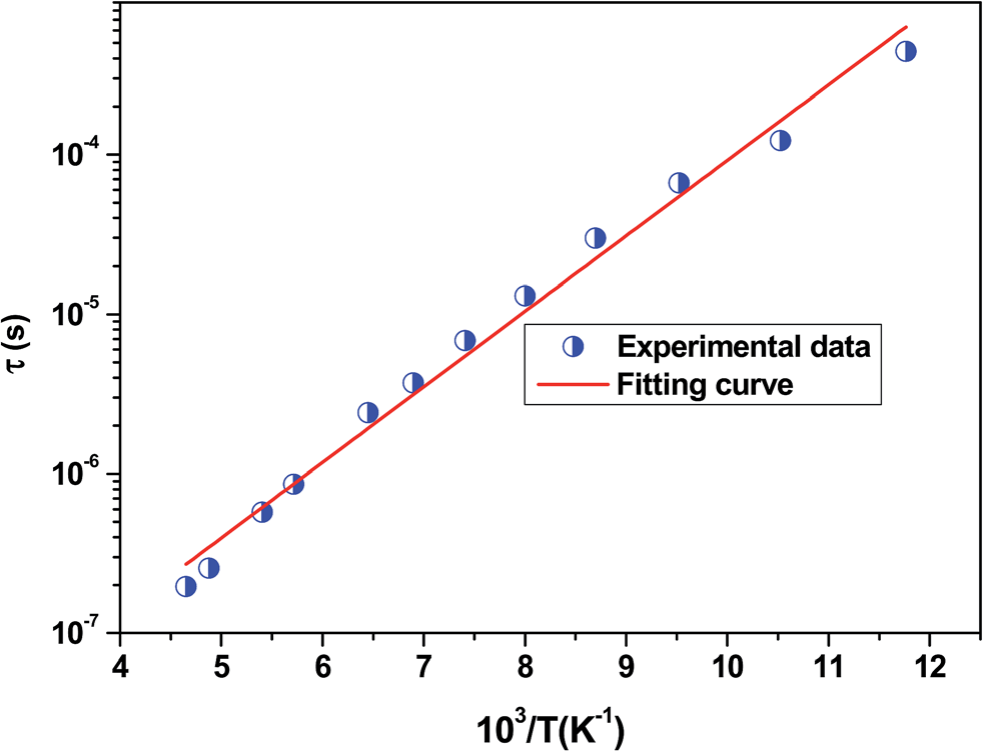
Variation of log(*τ*) *versus* 10^3^/*T* for the La_0.6_Gd_0.1_Sr_0.3_Mn_0.75_Si_0.25_O_3_ sample. Red solid line is the linear fit for our data.

The position of these peaks allows for the determination of the relaxation frequency value (*f*_max_) and the relaxation time (*τ*) using the relation: *τ*  =  1/2π *f*_max_. The variation of log(*τ*) *versus* 10^3^/*T* is shown in Fig. 11. We can see that the value of *τ* decreases with the increase in temperature, which suggests a thermally activated process. The dynamics of the relaxation process can be analyzed by the mean relaxation time *τ* expressed by the Arrhenius law 
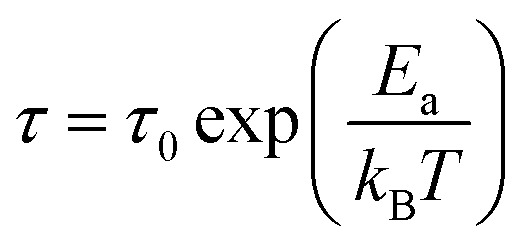
. This relaxation process is characterized by the activation energy (*E*_a_) and a relaxation time (*τ*_0_) of about 41.7 meV and 1.55 × 10^−9^ s, respectively. The *E*_a_ value is in good agreement with that deduced from the modulus analysis, *E*_relax_, which is about 42.92 meV. It is interesting to note that the energy deduced *E*_relax_ is equal to the activation energy *E*_a_, which signifies that the relaxation process and therefore the electrical conductivity, is attributed to the effects of the grain boundaries. Indeed, the modulus analysis paragraph has reveals that the dielectric relaxation is assigned to the grain boundary effect.

## 4. Conclusions

A La_0.6_Gd_0.1_Sr_0.3_Mn_0.75_Si_0.25_O_3_ ceramic was prepared by a solution-based sol–gel method, and its electrical properties were studied using the CIS technique. X-ray diffraction data confirmed that the sample was single phase with no detectable impurities and crystallized in the orthorhombic structure with the space group *Pnma*. The electrical properties were found to be strongly dependent on temperature and frequency. The ac conductivity spectra were found to obey the Jonscher's power law at different temperatures. The increase of the exponent, *n*, with the increasing temperature revealed a hopping process that occurred at longer distances for lower temperatures and between neighboring sites for higher temperatures. The impedance analysis revealed that low conductivity and high impedance values were observed at low temperatures. A broad peak observed in *Z*′′ *vs.* frequency plots at a particular temperature was sensitive to the concentration of silicon, and the peak shifts towards higher frequencies with the increasing temperatures demonstrated the multiple relaxation processes in the material. The relaxation time of dipoles was found to decrease with the increasing temperature.

## Conflicts of interest

There are no conflicts to declare.

## Supplementary Material
